# The Ubiquitin Ligase Adaptor NDFIP1 Interacts with TRESK and Negatively Regulates the Background K^+^ Current

**DOI:** 10.3390/ijms25168879

**Published:** 2024-08-15

**Authors:** Enikő Pergel, Dániel J. Tóth, Dóra Baukál, Irén Veres, Gábor Czirják

**Affiliations:** 1 Department of Physiology, Semmelweis University, 1094 Budapest, Hungary; pergel.eniko@semmelweis.hu (E.P.); baukal.dora@semmelweis.hu (D.B.); veres.iren@semmelweis.hu (I.V.); 2 HUN-REN-SU Molecular Physiology Research Group, Hungarian Research Network and Semmelweis University, 1094 Budapest, Hungary

**Keywords:** TRESK, K2P, K2P18, KCNK18, K^+^ channel, NDFIP, Nedd4, ubiquitin, (L/P) PxY, Xenopus oocyte

## Abstract

The TRESK (K2P18.1, KCNK18) background potassium channel is expressed in primary sensory neurons and has been reported to contribute to the regulation of pain sensations. In the present study, we examined the interaction of TRESK with NDFIP1 (Nedd4 family-interacting protein 1) in the *Xenopus* oocyte expression system by two-electrode voltage clamp and biochemical methods. We showed that the coexpression of NDFIP1 abolished the TRESK current under the condition where the other K^+^ channels were not affected. Mutations in the three PPxY motifs of NDFIP1, which are responsible for the interaction with the Nedd4 ubiquitin ligase, prevented a reduction in the TRESK current. Furthermore, the overexpression of a dominant-negative Nedd4 construct in the oocytes coexpressing TRESK with NDFIP1 partially reversed the down-modulating effect of the adaptor protein on the K^+^ current. The biochemical data were also consistent with the functional results. An interaction between epitope-tagged versions of TRESK and NDFIP1 was verified by co-immunoprecipitation experiments. The coexpression of NDFIP1 with TRESK induced the ubiquitination of the channel protein. Altogether, the results suggest that TRESK is directly controlled by and highly sensitive to the activation of the NDFIP1-Nedd4 system. The NDFIP1-mediated reduction in the TRESK component may induce depolarization, increase excitability, and attenuate the calcium dependence of the membrane potential by reducing the calcineurin-activated fraction in the ensemble background K^+^ current.

## 1. Introduction

TRESK (TWIK-related spinal cord K^+^ channel) is a unique member of the two-pore-domain (K_2P_) background potassium channel family [1,2]. TRESK alone represents a separate K_2P_ subfamily in vertebrate species. Its amino acid sequence shares a low (<20%) level of identity with other background K^+^ channels, and the similarity is mostly confined to transmembrane segments (TMS) [3,4,5]. TRESK shows a highly selective expression profile. In mammals, it is predominantly expressed in small- to medium-sized primary sensory neurons of the dorsal root (DRG) and trigeminal ganglia (TRG) [6,7], mainly corresponding to the MrgprD^+^ (Mas-related G-protein-coupled receptor member D positive) non-peptidergic and CGRP^+^ (calcitonin gene-related peptide positive) peptidergic nociceptors and low-threshold mechanoreceptor subpopulations [8,9]. The activation of TRESK and the resulting hyperpolarization reduced the excitability of nociceptive neurons and attenuated the pain sensation in various experimental models [10,11,12,13,14]. Accordingly, a role for TRESK in different pain disorders has been hypothesized and the channel has been reported as a putative pharmacological target in the treatment of neuropathic pain and migraines [15,16,17,18,19].

In contrast to other K_2P_ channels, the TRESK current is regulated by the cytoplasmic calcium concentration [4]. In response to the calcium signal, TRESK is robustly activated by the dephosphorylation of specific serine residues in the long cytoplasmic loop of the channel [20,21]. The dephosphorylation is mediated by the calcium-/calmodulin-dependent protein phosphatase calcineurin, which directly interacts with specific motifs in the channel [22,23]. In addition to *Xenopus* oocytes [24,25], calcium-dependent TRESK activation has been reported in DRG neurons [26], suprachiasmatic nucleus (SCN) neurons [27], hippocampal pyramidal neurons in epilepsy [28], and regulatory T cells [29]. In addition to physiological regulation, pharmacological TRESK modulators have also been identified. Cloxyquin is a state-dependent activator of TRESK, which exerts its effect under basal conditions, but not after calcium-dependent activation [30,31,32]. Although high (100 μM) concentrations are required, cloxyquin has also been used to activate TRESK in vivo [33,34,35]. Lamotrigine [36,37], sanshool [38], pyrethroids [39], and chemically modified derivatives of cloxyquin [40] were found to inhibit this channel.

While calcineurin and pharmacological compounds acutely influence the TRESK current, much less is known about the mechanisms of the long-term regulation of the channel. Here, we report that the ubiquitin ligase adaptor protein NDFIP1 functionally interacts with human TRESK. NDFIP1, Nedd4 family interacting protein 1, is a small, 221-amino acid protein with three TMSs that localizes to the Golgi, endosomes, and multivesicular bodies [41,42]. NDFIP1 associates to specific target proteins and contributes to their ubiquitination and proteasomal/lysosomal degradation [43,44,45,46,47]. The cytoplasmic N-terminal region of human NDFIP1 contains three (L/P) PxY motifs (where x corresponds to any amino acid in the single-letter code), also simply called PPxY or PY elements [41,48]. HECT-type E3 ubiquitin ligases such as Nedd4 (neural precursor cell-expressed, developmentally down-regulated 4) and Itch (itchy skin phenotype and multi-organ inflammation in knock-out mice) are recruited to NDFIP1 by direct protein—protein interactions [49,50]. The WW domains (named after two conserved tryptophans) of Nedd4 or Itch bind to PY motifs of NDFIP1; thus, the E3 ubiquitin ligases are released from autoinhibitory intramolecular interactions. The catalytic HECT (homologous to the E6-AP carboxyl terminus) domain of Nedd4 or Itch transfers ubiquitin to the target protein, resulting in mono-, multi-, or poly-ubiquitination. In the present study, we showed for the first time that the coexpression of NDFIP1 with TRESK in *Xenopus* oocytes results in the ubiquitination of the channel protein and significantly reduces the background K^+^ current.

## 2. Results

### 2.1. The Coexpression of NDFIP1 with TRESK Reduces the Background K^+^ Current

**Figure 1:**
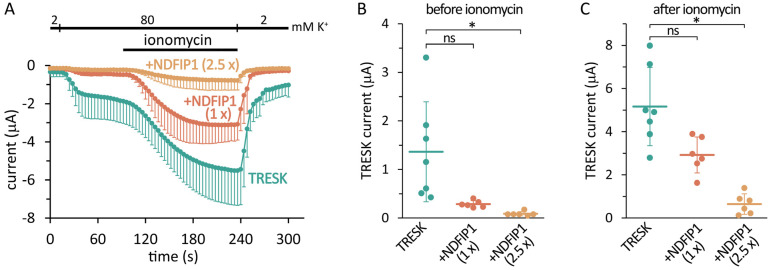
**NDFIP1 reduced the TRESK background K^+^ current.** (**A**) Two-electrode voltage clamp (TEVC) measurement of three groups of *Xenopus* oocytes expressing human *TRESK* (*n* = 7), coexpressing TRESK with human NDFIP1 (*+NDFIP1* (1×), *n* = 6), or coexpressing the channel with a higher amount of NDFIP1 (*+NDFIP1* (2.5×), *n* = 6; two and half times more NDFIP1 cRNA was microinjected). Inward (negative) currents were measured at −100 mV. Extracellular [K^+^] was increased from 2 to 80 mM, as shown *above the curves*, and the oocyte was then challenged with *ionomycin* (0.5 μM), as indicated by the *horizontal black bar*. The average currents and SD are plotted. (**B**) Statistical analysis of the effect of NDFIP1 on the basal TRESK current. The basal TRESK currents were calculated as the difference in 2 and 80 mM [K^+^], before the application of ionomycin (see panel (**A**)). (**C**) Statistical analysis of the effect of NDFIP1 on the TRESK current stimulated with ionomycin. The stimulated TRESK currents were measured at the end of the application of ionomycin in 80 mM [K^+^] (see panel (**A**)), and the small currents in 2 mM [K^+^] were subtracted. * *p* < 5 × 10^−4^, ns: not significant (Dunn’s test after Kruskal–Wallis ANOVA). Although not significant with Dunn’s test, the difference in the basal currents between the *TRESK* and *NDFIP1* (1×) groups in panel B was significant with the Mann–Whitney U test (*p* < 0.005), which gave *p* < 0.015 after Bonferroni’s correction. *TRESK* vs. *NDFIP1* (1×) in panel (**C**) gave *p* < 0.05 with the Mann–Whitney U test after Bonferroni’s correction.

TRESK and different amounts of NDFIP1 cRNAs were coinjected into *Xenopus laevis* oocytes, and the resulting background potassium currents were examined three days later (Figure 1A). The currents were measured by the two-electrode voltage clamp method at −100 mV, and the basal TRESK current was calculated as a difference in extracellular solutions containing 2 or 80 mM [K^+^]. Since TRESK is typically regulated by calcineurin, the calcium ionophore ionomycin was applied during the measurement to increase cytoplasmic [Ca^2+^], stimulate the endogenous calcium-/calmodulin-dependent protein phosphatase, and activate TRESK by dephosphorylation [4,22,23].

The coexpression of NDFIP1 dose-dependently decreased both the basal and stimulated TRESK currents (Figure 1A). As an advantage of the *Xenopus* oocyte system, the NDFIP1 expression level could be adjusted by changing the amount of microinjected cRNA, and the functional effect of NDFIP1 on TRESK was examined. When a sufficiently high NDFIP1 expression was induced, the TRESK current was abolished, and the effect reached statistical significance with the Kruskal–Wallis test (Figure 1B,C).

**Figure 2:**
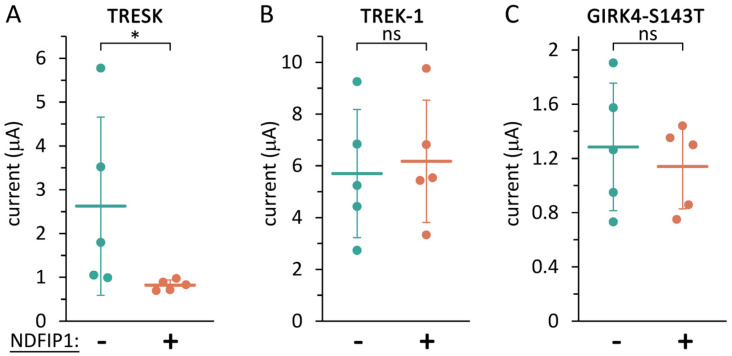
**TRESK was regulated by NDFIP1 more effectively than TREK-1 and GIRK4.** (**A**) K^+^ currents in two groups of oocytes expressing TRESK, or coexpressing TRESK with NDFIP1 (0.67×), as indicated *below the plot*. (**B**) Similar experiment as in panel A with TREK-1. (**C**) Similar experiment as in panel A with mutant GIRK4. Data are from the same oocyte preparation, *n* = 5 in all groups. * *p* < 0.01 with Mann–Whitney U test, ns: not significant.

The reduction of the TRESK current by NDFIP1 suggests that there is a functional interaction between the two proteins. We asked whether this interaction is specific, since the overexpression of NDFIP1 was previously shown to disrupt the Golgi apparatus and interfere with the overall protein expression [41]. Therefore, we also tested the effect of NDFIP1 on two other potassium channel types under identical conditions, on the relatively closely related K_2P_ channel TREK-1 (TWIK-related K^+^ channel 1, K_2P_2.1, *KCNK2*), and on a more distantly related K^+^ channel, GIRK4 (G protein-activated inward rectifier potassium channel 4, K_IR_3.4, *KCNJ5*). We used the S143T mutant version of GIRK4, as it can form functional homotetramers even in the absence of other GIRK subunits [51]. Our approach was to adjust the NDFIP1 expression to a level that inhibited TRESK, but did not affect TREK-1 or GIRK4. When the amount of NDFIP1 cRNA was two thirds of the quantity used in Figure 1, this microinjection was sufficient to significantly reduce the TRESK current (Figure 2A); however, TREK-1 and GIRK4 were not affected in the same oocyte preparation under identical conditions (Figure 2B,C). Therefore, it was possible to establish an appropriate NDFIP1 expression level for the specific regulation of TRESK, indicating that TRESK is more sensitive to NDFIP1 than TREK-1 and GIRK4. The TRESK current was reduced by a relatively low level of NDFIP1, which did not generally interfere with the functional expression of other channel proteins. In the following experiments, this amount of NDFIP1 cRNA (0.67×) was used.

### 2.2. The Effect of NDFIP1 on TRESK Is Mediated by E3 Ubiquitin Ligases

**Figure 3:**
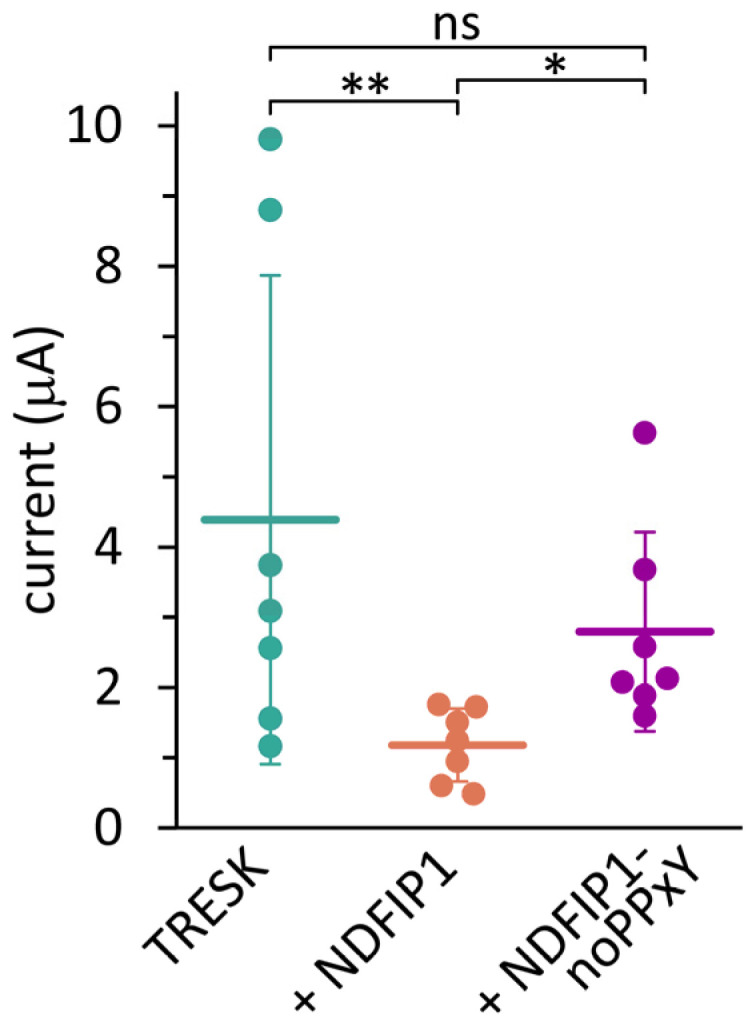
**Mutation of PY motifs in NDFIP1 prevented the effect of the adaptor protein on TRESK.** K^+^ currents in three groups of oocytes expressing *TRESK*, coexpressing TRESK with *NDFIP1*, or coexpressing TRESK with a mutant version of NDFIP1 lacking PY motifs (*NDFIP1-noPPxY*), as indicated *below the plot*. Data are from the same oocyte preparation, *n* = 7 in all groups. * *p* < 0.04, ** *p* < 0.02 with Dunn’s test after Kruskal–Wallis ANOVA, ns: not significant.

The three PY elements of human NDFIP1 were mutated (see the Methods section) in order to prevent the binding of E3 ubiquitin ligases to the adaptor protein. The effect of this NDFIP1-noPPxY construct on TRESK was compared with wild-type NDFIP1 in the same oocyte preparation (Figure 3). The wild-type NDFIP1 reduced the TRESK current as expected, but the mutant NDFIP1-noPPxY protein did not significantly alter the background K^+^ current. This result is consistent with the hypothesis that NDFIP1 recruits E3 ligases through PY motifs to ubiquitinate the TRESK protein.

**Figure 4:**
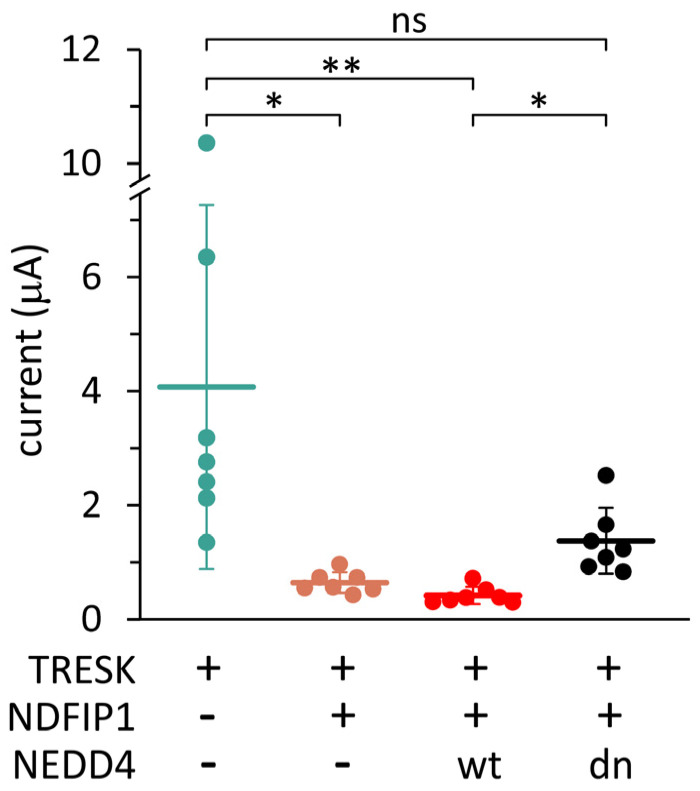
**Dominant negative Nedd4 counteracted the NDFIP1-mediated reduction in the TRESK current.** K^+^ currents in four groups of oocytes expressing TRESK, coexpressing TRESK with NDFIP1, or coexpressing wild-type (*wt*) or dominant negative (*dn*) Nedd4 with TRESK and NDFIP1 (triple coexpression), as indicated *below the plot*. Data are from the same oocyte preparation, *n* = 7 in all groups. * *p* < 0.015, ** *p* < 10^−4^ with Dunn’s test after Kruskal–Wallis ANOVA, ns: not significant.

To verify the role of E3 ubiquitin ligases in TRESK regulation, another experimental approach was applied. We cloned human Nedd4 and also produced a dominant negative variant of this E3 ligase by mutating the catalytic HECT domain (see the Methods). We assumed that the intact WW domains of this inactive Nedd4 construct occupied the PY motifs of NDFIP1; however, the mutant could not ubiquitinate TRESK. Wild-type or dominant negative Nedd4 was coexpressed with TRESK and NDFIP1 (triple coexpression, Figure 4).

Wild-type Nedd4 reduced the TRESK current to very low levels in the presence of NDFIP1 (see the group coexpresssing *wt* Nedd4 in Figure 4). In sharp contrast, NDFIP1 did not reduce the K^+^ current significantly in the presence of dominant negative Nedd4 (*dn* Nedd4 in Figure 4, compared to the cells expressing only the channel). The only difference between the groups expressing *wt* or *dn* Nedd4 was a single point mutation in the ubiquitin ligase. Therefore, the significantly different K^+^ currents of the two groups suggest that Nedd4 regulates TRESK.

### 2.3. TRESK Regulation by NDFIP1 Does Not Depend on the Ubiquitination of a Single Lysine Residue in the Channel

**Figure 5:**
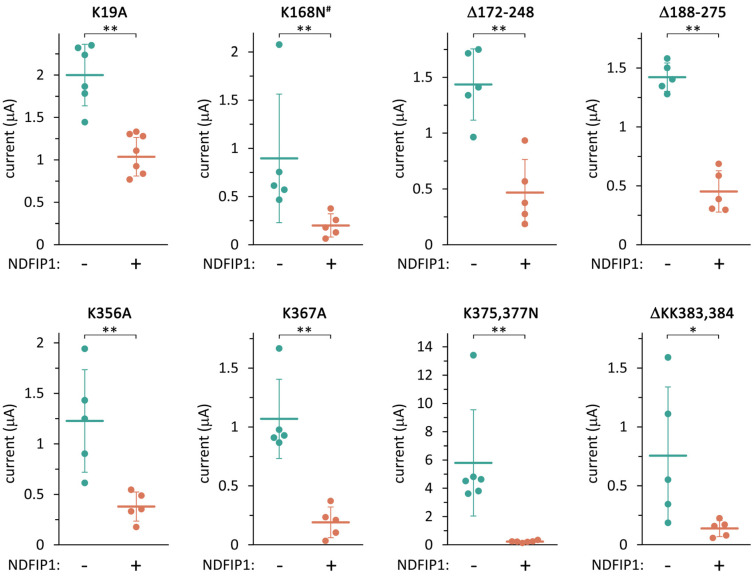
**NDFIP1 reduced the K^+^ current of all the tested lysine-mutant TRESK constructs.** The K^+^ currents in the oocytes expressing the different lysine-mutant TRESK constructs (see *above the panels*), in the absence or presence of NDFIP1 coexpression, as indicated *below the graphs*. Data points on the same panel always come from the same oocyte preparation, *n* = 5–7 in all groups. * *p* < 0.02, ** *p* < 0.01 with Mann–Whitney U test. ^#^ RFRK motif of TRESK was mutated to NFNN, including the K168N mutation. In the Δ172–248 and Δ188–275 constructs, the appropriate regions were replaced by a lysine-free linker. In the ΔKK383,384 construct, the C-terminal double lysine was deleted.

We would have liked to identify the target lysine residue of the TRESK ubiquitination. Therefore, we investigated the effect of NDFIP1 on a series of mutant TRESK channels (Figure 5). Human TRESK contains 16 lysine residues in its intracellular regions. Some lysine residues were mutated individually or in combination with an adjacent lysine. Some other lysines were removed in constructs where large regions of TRESK were replaced with a flexible polypeptide linker (see the Methods section). The Δ172–248 and Δ188–275 constructs did not contain eight and four lysines, respectively, in the long intracellular loop of TRESK.

NDFIP1 significantly reduced the K^+^ current of all the lysine-mutant constructs, suggesting that no single lysine residue was uniquely responsible for the regulatory mechanism. The data also excluded a combined contribution of the eight lysines between residues 172 and 248 of the intracellular loop. The suspicion that the K19A mutation mitigates the effect of NDFIP1 has not reached statistical significance with the high number of compared constructs. The results are compatible with combinations of ubiquitination, including residues outside the 172–248 region, or other auxiliary mechanisms of NDFIP1 action on TRESK. The effect of NDFIP1 could not be abolished by any of the TRESK mutations, suggesting that redundant mechanisms ensure the regulation of the channel by the adaptor protein.

### 2.4. TRESK Is Ubiquitinated When NDFIP1 Is Coexpressed

**Figure 6:**
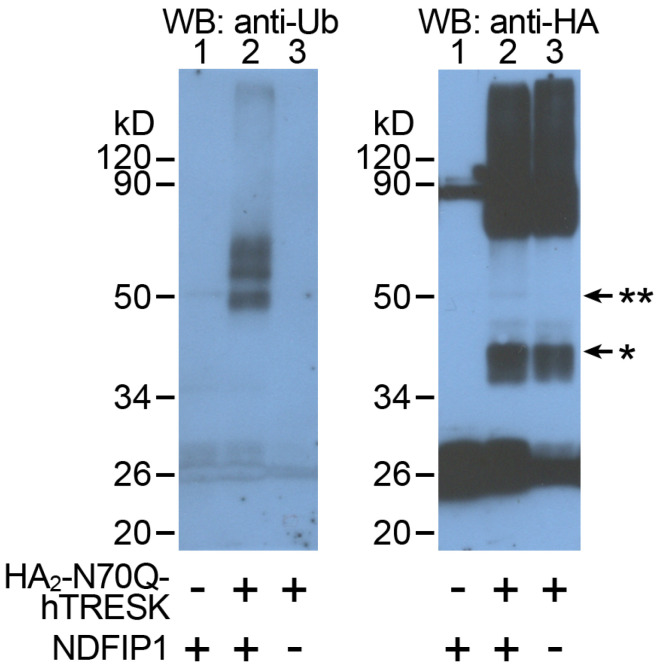
**Human TRESK was ubiquitinated in an NDFIP1-dependent manner.** The HA_2_-N70Q-hTRESK protein was pulled down with anti-HA resin from the solubilized membrane preparations of three groups of oocytes expressing HA-tagged TRESK or NDFIP1, or coexpressing both proteins, as indicated *below the images*. On the *left side*, a Western blot was performed with an anti-ubiquitin primary antibody (*anti-Ub*, as indicated *above the image*). On the *right side*, an anti-HA immunoblot of the same membrane is shown. Representative of three similar experiments. * monomeric TRESK protein, ** mono-ubiquitinated TRESK in lane 2.

The ubiquitination of the TRESK protein was examined by expressing HA_2_-N70Q-hTRESK in *Xenopus* oocytes. This construct contained two influenza hemagglutinin epitope (HA) tags on the intracellular N-terminus of the channel, and the N70Q mutation was introduced to prevent N-glycosylation and heterogeneous migration on SDS-PAGE gels [52,53]. HA_2_-N70Q-hTRESK was expressed alone or coexpressed with NDFIP1, or, in a third control group of oocytes, only NDFIP1 was expressed. Crude membrane preparations were produced from these three groups of oocytes by centrifugation (see the Methods section). Afterwards, the phospholipid membranes were solubilized by NP-40 detergent and the HA_2_-N70Q-hTRESK protein was pulled down with the anti-HA antibody-coated resin of Pierce^TM^ HA-Tag IP/Co-IP Kit. The ubiquitination of the channel protein was detected by a standard immunoblot technique, and the same blotting membrane was stripped and reprobed with anti-ubiquitin antibodies after an anti-HA Western blot (Figure 6).

Ubiquitinated bands of the TRESK protein are clearly visible in lane 2 of the anti-ubiquitin immunoblot. The covalent binding of ubiquitin, which consists of 76 amino acids, increases the molecular weight of the monomeric TRESK protein by about 8.6 kD. In addition to the mono-ubiquitinated TRESK protein around the 50 kD molecular weight marker, two additional bands were detected, corresponding to the association of two or three ubiquitin units to the channel. Accordingly, TRESK was multi- or poly-ubiquitinated in response to the coexpression of NDFIP1. These bands were completely absent from the control groups, indicating that the ubiquitination of TRESK was negligible in the absence of NDFIP1, and that the TRESK signal was specific. The anti-HA immunoblot also faintly showed the most abundant mono-ubiquitinated TRESK component in *lane 2* (Figure 6, *right side*). Ubiquitination was not detected in dimeric and higher-order aggregates of TRESK, which were not well resolved by the anti-HA blot. Altogether, these data clearly support the conclusion that the TRESK protein is ubiquitinated.

### 2.5. Protein–Protein Interaction between TRESK and NDFIP1

**Figure 7:**
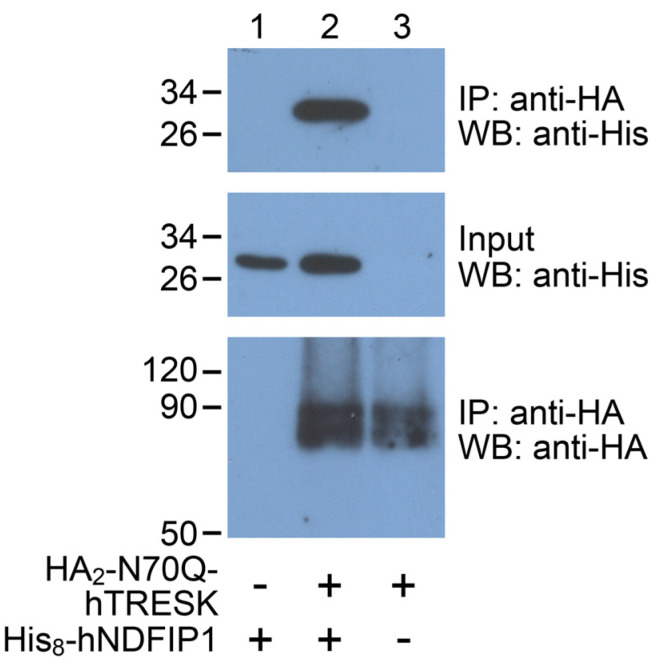
**NDFIP1 is co-immunoprecipitated with TRESK.** Three groups of oocytes expressed HA_2_-N70Q-hTRESK, His_8_-hNDFIP1, or both proteins, as indicated *below the image*. The HA_2_-N70Q-hTRESK protein was pulled down with anti-HA resin from the solubilized membrane preparations of these oocyte groups, and the co-immunoprecipitation of His_8_-hNDFIP1 was detected with an anti-His immunoblot (*upper panel*). Aliquots of the solubilized membrane preparations (*Input*) were also analyzed with an anti-His immunoblot on the same blotting membrane (*middle panel*). The presence of the bait protein was verified in the samples after anti-HA immunoprecipitation with the anti-HA immunoblot (*lower panel*; dimeric TRESK was detected). Representative of three similar experiments. IP: immunoprecipitation; WB: Western blot/immunoblot; anti-HA: antibody against the influenza hemagglutinin epitope; anti-His: antibody against the hexahistidine tag.

An interaction between TRESK and NDFIP1 was first predicted by high-throughput screening of the entire human interactome, which resulted in 118,162 interactions among 14,586 proteins in 293T cells [54]. Although high-throughput screening methods may result in false positive hits, the above functional and biochemical data are in favor of the hypothesis that this interaction is real. Nevertheless, we decided to verify the interaction of TRESK with NDFIP1 by co-immunoprecipitation in our applied experimental system, in *Xenopus* oocytes (Figure 7).

An octahistidine tag was fused to the N-terminus of human NDFIP1, and the interaction of this His_8_-hNDFIP1 protein with HA_2_-N70Q-hTRESK was examined. The anti-HA resin pulled down His_8_-hNDFIP1 only in the presence of HA_2_-N70Q-hTRESK (see *lane 2* in the *upper panel* of Figure 7), indicating that NDFIP1 was associated with TRESK in the oocytes coexpressing both proteins. This association may be a direct physical interaction or may be mediated indirectly by unknown endogenous components of the oocyte. In the absence of HA_2_-N70Q-hTRESK, no His_8_-hNDFIP1 was recovered after the anti-HA immunoprecipitation (*lane 1*, *upper panel*), although His_8_-hNDFIP1 was present in the input before the pull-down reaction (*lane 1*, *middle panel*). This excludes the possibility of a non-specific interaction between the anti-HA resin and the NDFIP1 protein. In the absence of His_8_-hNDFIP1, the pull-down of HA_2_-N70Q-hTRESK (*lane 3*, *lower panel*) did not result in a visible band in the anti-His immunoblot (*lane 3*, *upper panel*), verifying the specific detection of His_8_-hNDFIP1 with the anti-His antibody.

### 2.6. Coexpression of NDFIP1 Reduces the Quantity of TRESK Protein on the Oocyte Surface

**Figure 8:**
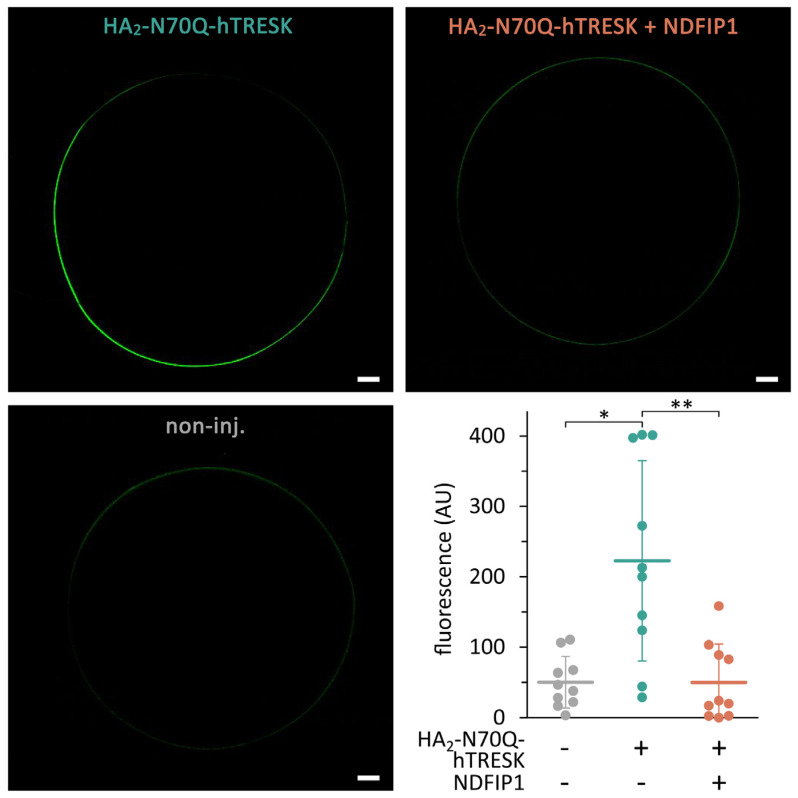
**Coexpression of NDFIP1 decreased the immunofluorescence signal of HA-tagged TRESK on the oocyte surface.** Three groups of oocytes expressed HA_2_-N70Q-hTRESK, coexpressed this construct with NDFIP1, or remained as a non-injected control (*non-inj*), as indicated in the *representative images* and *below the graph*. *White scale bars* indicate 100 μM. Data are from the same oocyte preparation, *n* = 10 in all groups. * *p* < 0.02, ** *p* < 0.005 with Dunn’s test after Kruskal–Wallis ANOVA.

Fluorescent immunolabeling and confocal microscopy were used to estimate the amount of TRESK protein on the surface of fixed and permeabilized oocytes. The HA_2_-N70Q-hTRESK construct was visualized by indirect immunofluorescence using an anti-HA primary antibody and a secondary antibody conjugated with Alexa Fluor 488 fluorescent dye (Figure 8).

In good accordance with the electrophysiological data, the coexpression of NDFIP1 decreased the quantity of the TRESK protein in the plasma membrane and/or in the membranes of intracellular compartments located in the vicinity of the cell surface. This suggests that the decreased number of channels contributes to the reduction in the background K^+^ current.

## 3. Discussion

The straightforward interpretation of the results is that NDFIP1 binds to TRESK via a protein–protein interaction, Nedd4 is recruited to the PY motifs of NDFIP1 and ubiquitinates TRESK, and, therefore, the channel is targeted for degradation, causing a reduction in the background K^+^ current through the plasma membrane. All elements of this summarizing conclusion on the novel mechanism of long-term TRESK regulation require further discussion.

Both TRESK and NDFIP1 are integral membrane proteins; thus, their interaction may occur in phospholipid bilayers. The mechanism of interaction between NDFIP1 and its partner proteins is not fully understood, but it has been suggested that Bsd2 (bypass Sod1p defect 2), the yeast ortholog of NDFIP1, recognizes polar transmembrane segments in its target proteins (i.e., TMSs containing hydrophilic residues) [48]. There is a consensus in the literature that NDFIP1 is predominantly located in membranes of the Golgi apparatus [41,55]. Therefore, it is most likely that NDFIP1 interacts with TRESK at this site, when the TRESK protein travels through and matures in the Golgi complex. In fact, there are two similar NDFIP proteins in mammals, NDFIP1 and NDFIP2 (these were previously named N4WBP5 and N4WBP5A, respectively). Although NDFIP2 was not examined in this study, based on the high sequence similarity and high-throughput interaction screening data, NDFIP2 may also interact with TRESK [54]. NDFIP2 has been shown to localize mainly in the multivesicular body (MVB) and late endosomal vesicles in addition to the Golgi apparatus [55,56]. Therefore, another possible location for the regulation of TRESK by NDFIP proteins is the endocytic pathway.

We used wild-type and dominant negative Nedd4 to investigate the role of ubiquitin ligases in NDFIP1-dependent TRESK regulation. However, other HECT-type E3 ubiquitin ligases may also bind to NDFIP proteins. There are nine members of the Nedd4 subfamily of HECT-type E3 ubiquitin ligases: Nedd4 (Nedd4-1), Nedd4-2, Itch, Smurf1 (Smad ubiquitination regulatory factor 1), Smurf 2, WWP1 (WW-domain-containing E3 ubiquitin protein ligase 1), WWP2, HECW1 (HECT, C2, and WW-domain-containing protein 1), and HECW2 [50]. Each of these contains multiple (2–4) WW domains that can potentially bind to the PY motifs of NDFIP1 [42]. For example, NDFIP1 has been shown to interact with Nedd4 [57], Nedd4-2 [44], Itch [43], Smurf1 [58], or WWP2 [45] in different cell types and under different experimental settings. Therefore, TRESK may also be ubiquitinated by other Nedd4 class HECT-type E3 ubiquitin ligases besides Nedd4 in different native cells.

The experimental data clearly indicate that TRESK is ubiquitinated by endogenous ubiquitin ligases upon NDFIP1 coexpression in *Xenopus* oocytes. It appears that the effect of NDFIP1 on TRESK is specific in the sense that the TREK-1 and GIRK4 channels are not affected by the coexpression of the adaptor protein under the same conditions. The specific regulation of other target proteins by NDFIPs has been clearly established in the literature, although Bsd2, the only NDFIP ortholog in yeast, has also been suggested to contribute to the general elimination of misfolded transmembrane proteins [48]. Among the best-studied examples of specific targets of NDFIP1 is divalent metal transporter 1 (DMT1), which plays an important role in nonheme iron absorption. NDFIP1 knock-out mice show alterations in iron homeostasis [45]. This relationship appears to be evolutionarily conserved, since mutations in Bsd2 in yeast cause the hyperaccumulation of heavy metal ions [59], and metal toxicity in human neurons has also been found to be related to NDFIP1 expression, e.g., in the case of metal implants [44,60].

Another extensively investigated target protein of NDFIP-dependent regulation is the transcription factor JunB in T lymphocytes (for review, see [61]). The NDFIP1-dependent activation of the Itch ubiquitin ligase results in the degradation of JunB, thereby limiting interleukin-4 (IL-4) production from T helper type 2 (TH2) cells. A knock-out of NDFIP1 or Itch in mice leads to severe inflammatory disease, and mutations in human Itch are linked to a rare autoimmune disorder [43,62]. In the context of TRESK, it is interesting that the transforming growth factor-β (TGF-β)-induced transient NDFIP1 expression has been suggested to utilize the NDFIP1/Itch/JunB sequence during regulatory T cell (Treg) development [63,64]. This system has complex temporal and Treg type-dependent characteristics. As an apparently independent observation, another group of researchers recently reported that the genetic or pharmacological manipulation of the TRESK expression or function influences thymic Treg differentiation [29]. Attempting to fit the conclusion of our present study as a missing puzzle piece between the two previously reported results would give rise to the hypothesis that a TGF-β/NDFIP1/Itch/TRESK pathway may exist to modulate regulatory T cell development and function.

There are also other well-characterized specific targets of NDFIPs, such as the tumor suppressor phosphatidylinositol-3,4,5-trisphosphate (PIP_3_) 3-phosphatase PTEN (phosphatase and tensin homolog) [57,65], and one example of regulated proteins was even reported among the potassium channels. The hERG (human ether-a-go-go-related gene, Kv11.1, *KCNH2*) K^+^ channel, responsible for the IKr (rapid) current component in cardiac myocytes and mutated in type 2 long-QT syndrome, was reported to interact with NDFIP1 and NDFIP2 and was ubiquitinated by Nedd4-2 [55]. The hERG channel is a voltage-gated K^+^ channel; accordingly, it has a molecular architecture of 6TMS/1P, i.e., six transmembrane segments and one pore domain in a subunit, and forms functional channels as a homotetramer. In contrast, TRESK belongs to another K^+^ channel family; it is a K_2P_ background channel with 4TMS/2P architecture, which constitutes the functional dimers of the subunits. The regulation of hERG by Nedd4-2 also appears to be different from TRESK, since the C-terminal region of the hERG subunit contains a functional PY element. The four PY elements of the hERG tetramer play an important role in the direct localization of Nedd4-2 ubiquitin ligase, in addition to the interaction of hERG with NDFIPs. TRESK does not contain an (L/P) PxY motif; therefore, its ubiquitination appears to be dependent on the association of NDFIPs to the channel protein.

In the present study, we provided evidence that TRESK is ubiquitinated, the amount of TRESK protein decreases on the cell surface, and the background K^+^ current through the plasma membrane is reduced. As a parsimonious interpretation, we deduce that TRESK is targeted for degradation, although our data do not formally exclude the alternative hypothesis that TRESK expression is reduced. We think that ubiquitination is a well-established signal for protein degradation; therefore, this explanation is more likely than the possible inhibition of cRNA translation by NDFIPs, especially considering that the TREK-1 and GIRK4 currents are not affected under identical conditions. Furthermore, NDFIP1 may reduce the TRESK current by acutely inhibiting the channel. The binding of NDFIP1 to TRESK (directly or indirectly), or the consequent ubiquitination of the channel protein, may exert an inhibitory effect and reduce the K^+^ current independently of the alterations in the number of channels. The exact ratio between the two mechanisms could not be determined from our results. While the reduction in the number of channels is probable, additional acute channel inhibition remains a feasible possibility.

Ubiquitination often results in proteasomal degradation; however, ubiquitinated transmembrane proteins in the Golgi apparatus or multivesicular body (MVB) are rather processed in the lysosome [66]. Vesicular trafficking between the trans-Golgi network and the endosome/lysosome system is mediated by GGA (Golgi-localized, gamma adaptin ear domain homology, ARF-binding) coat proteins, which specifically recognize the ubiquitinated cargo [67]. Accordingly, in the example of NDFIP-dependent hERG regulation, it has been postulated that NDFIP1 interacts with hERG in the Golgi complex, and then the channel is transported to the MVB and, from there, to lysosomal degradation [55]. However, another NDFIP-interacting protein, Robo1, has been suggested to be degraded by both proteasomal and lysosomal mechanisms in response to NDFIP1 expression, whereas it is mostly eliminated in the lysosome when NDFIP2 evokes the effect [68]. The detailed elucidation of the intracellular trafficking of the TRESK protein, whether it is degraded in the proteasome or lysosome, will require a different methodology in further studies on other expression systems.

It is widely accepted in the literature that TRESK is expressed in specific subpopulations of primary sensory neurons in the dorsal root and trigeminal ganglia (DRG and TRG) [6,7,12,17,18]. The function of NDFIP1 and NDFIP2 has not yet been directly investigated in these neurons; however, single-cell RNA sequencing data show that both NDFIPs are abundantly expressed in different sensory neuron types in DRG [8] and TRG [9]. According to the results of the present study, the regulation of TRESK by ubiquitination could be considered in all cell types coexpressing this background K^+^ channel with NDFIP adaptor proteins. The reduction in the number of TRESK channels in the plasma membrane may result in a decrease of the background K^+^ conductance, and induce depolarization or increase excitability in response to other depolarizing stimuli.

Even if the resting membrane potential is restored by the compensatory overexpression of other K^+^ or Cl^−^ channels, the dependence of the membrane potential on the cytoplasmic calcium concentration may be reduced in the absence of TRESK. The calcineurin-dependent dephosphorylation and activation of TRESK in response to the calcium signal exerts a hyperpolarizing/repolarizing (or stabilizing) effect on the (resting) membrane potential [4,26,27,28,29]. The long-term decoupling of the membrane potential from cytoplasmic calcium by NDFIP1-mediated TRESK degradation may induce cell type- and context-dependent functional consequences in the detection of painful stimuli and possibly in the differentiation of regulatory T lymphocytes.

## 4. Materials and Methods

### 4.1. Materials

Chemicals of analytical grade were purchased from Merck/Sigma-Aldrich (Burlington, MA, USA), Enzo Life Sciences (Farmingdale, NY, USA), Santa Cruz Biotechnology (Dallas, TX, USA), Cayman Chemicals (Ann Arbor, MI, USA), or MedChemExpress (Monmouth Junction, NJ, USA). The enzymes and kits for molecular biology applications were purchased from Thermo Fisher Scientific (Waltham, MA, USA), iNtRON Biotechnology (Seongnam, Republic of Korea), Ambion (Austin, TX, USA), or QIAGEN (Hilden, Germany). Ionomycin was prepared as a stock solution in DMSO (5 mM). All the other chemicals were dissolved in DMSO or water, as recommended by the manufacturer. The stock solutions were stored at −20 °C and diluted further before the application.

### 4.2. Molecular Biology

The cloning of human TRESK cDNA (GenBank: *NM_181840.1*) has been previously described [4]. Human NDFIP1 (*NM_030571.4*) and Nedd4 (*BC136605.1*) cDNAs were amplified by PCR using Phusion polymerase (Thermo Fisher Scientific) after the reverse transcription (RevertAid Reverse Transcriptase, Thermo Fisher Scientific) of the total RNA purified from a human embryonal kidney HEK293 cell line with the TRIzol reagent (Invitrogen, Carlsbad, CA, USA). The Nedd4 clone contained the I863T amino acid substitution deviating from *BC136605.1*, but corresponding to *NM_001284338.2*. The 5′ *GAATTCGCCGCCACC* 3′ sequence, including an EcoRI restriction enzyme site and a Kozak sequence, was inserted before the start codon, the 5′ *CTCGAG* 3′ XhoI site was added directly after the stop codon, and NDFIP1 and Nedd4 were subcloned into the pXEN vector (*EU267939.1*) between the EcoRI and XhoI sites.

The K19A, K168N (RFRK motif to NFNN), K356A, K367A, “K375,377N”, and “ΔKK383,384” mutations in human TRESK were produced by the QuikChange in vitro site-directed mutagenesis method (developed by Stratagene, La Jolla, CA, USA), or with other PCR-based techniques using custom-designed oligonucleotides and Phusion polymerase. In the Δ172–248 and Δ188–275 TRESK constructs, the deleted amino acids were replaced with a 60-residue long poly-glutamine-containing linker: [LEHQ_9_]_5_. The preparation of HA_2_-N70Q-hTRESK has been previously described [52]. The NDFIP1-noPPxY construct was produced by mutating the PPPY and PPSY sequences to PPAG and the LPSY sequence to LPAG, as previously described [42]. In His_8_-hNDFIP1, the eight histidines were inserted after the start methionine. The dominant negative Nedd4 construct was obtained by introducing the C1286S mutation to eliminate the catalytic cysteine from the HECT domain, as previously reported [69,70]. The sequence of the clones was verified by the Sanger sequencing service of Microsynth (Vienna, Austria).

The cRNAs coding for the different TRESK, NDFIP1, and Nedd4 constructs were synthesized using the mMESSAGE mMACHINE™ T7 in vitro transcription kit (Ambion, Austin, TX, USA), according to the instructions of the manufacturer. The plasmid templates for the reaction were linearized with the XbaI, NotI, or SmaI restriction enzymes. Different dilutions of the same NDFIP1 cRNA preparation were used in all of the shown experiments, and the relative concentrations are given in parentheses in the figures, e.g., (2.5×) represents more, whereas (0.67×) represents less injected amount than the (1×) reference solution. The (1×) solution was a 100-fold dilution of the product of the synthesis, when the product of a 20 μL synthesis reaction was dissolved in 40 μL water after LiCl precipitation (see the instruction manual of the kit). The quality of all cRNA preparations was verified by denaturing formaldehyde agarose gel electrophoresis and ethidium bromide staining.

### 4.3. Two-Electrode Voltage Clamp

*Xenopus* oocytes were prepared, microinjected, and measured by the two-electrode voltage clamp method, as previously described [71,72]. The cells were incubated in modified Barth’s saline containing 1 mM [K^+^] [71], and the measurements were performed three or four days after the microinjection of cRNA. During the measurement, the low [K^+^] solution (indicated as “2 mM K^+^”) contained the following (in mM): NaCl: 95.4, KCl: 2, CaCl_2_: 1.8, HEPES 5 (pH 7.5 adjusted with NaOH). The high [K^+^] solution contained 80 mM K^+^ (78 mM Na^+^ of the low [K^+^] solution was replaced with K^+^). The K^+^ currents were measured at the end of the 300 ms long voltage steps to −100 mV applied in every 4 s.

### 4.4. (Co-)Immunoprecipitation

The HA_2_-N70Q-hTRESK, NDFIP1, and His_8_-hNDFIP1 constructs were expressed in *Xenopus* oocytes as indicated in the figure legends. Within the same experiment, each group contained an identical number of oocytes, 20–40 cells in the different experiments. Three days after the cRNA injection, the groups of oocytes were homogenized in 1 mL of ice-cold lysis solution with 20 strokes in a glass Potter homogenizer. The lysis solution contained the following: HEPES (20 mM), NaCl (80 mM), EDTA (10 mM), phenylmethylsulfonyl fluoride (PMSF, 5 mM), benzamidine (1 mM), leupeptine (5 μg/mL), and a pH of 7.2 (adjusted with NaOH). In the experiments to detect the ubiquitination of the TRESK protein, the cells were treated with 30 μM MG132 proteasome inhibitor (Thermo Scientific) for one day before homogenization, and the lysis solution was supplemented with the PR-619 deubiquitinase inhibitor (25 μM, Thermo Scientific).

The homogenates were centrifuged twice at 1000× *g* for 10 min at 4 °C in order to remove yolk granules, nuclei, and the floating lipid layer. Finally, the supernatant was centrifuged at 16,000× *g* for 10 min at 4 °C, and the crude membrane fraction was solubilized in a 200 μL lysis solution supplemented with 1% NP-40 detergent. An amount of 20 μL was saved as the “Input” for the control experiments, and the remaining 180 μL was applied on a Pierce Spin column (Pierce^®^ HA Tag IP/Co-IP Kit, Cat. #26180, Thermo Fisher Scientific). Anti-HA agarose (20 μL of a 50% suspension from the kit) was added to the column, mixed, and incubated overnight with a gentle rocking motion at 4 °C. The resin was pelleted on the column with a short (10 s) centrifugation, the flow-through was discarded, and the resin was washed three times with a 500 μL solution containing the following: Tris (25 mM, pH of 7.2, with HCl), NaCl (150 mM), and Tween-20 detergent (0.05%). Depending on the number of oocytes, the resin was resuspended in 40–80 μL of SDS loading buffer containing: Tris (0.31 M, pH of 6.8, with HCl), SDS (10%), glycerol (50%), mercaptoethanol (25%), and bromphenol blue (0.2%), and incubated for 20 min at room temperature. The eluate was recovered from the column by a short centrifugation and stored at −20 °C.

### 4.5. Western Blot

All the immunoblot experiments (with the exception of the control “Input” samples) were performed after the above-described (co-)immunoprecipitation step, i.e., after the immunoaffinity purification (“pull-down”) of the HA-epitope-tagged TRESK and the associated proteins from the crude membrane fraction of *Xenopus* oocytes. Five to fifteen μL denatured samples for each group were separated by SDS-PAGE on 10% gels and transferred to nitrocellulose membranes (Amersham Protan^TM^ 0.2 μm NC, GE Healthcare Life Sciences). The transfer (tank blotting) was performed overnight at 50 V in Towbin buffer containing 25 mM Tris, 192 mM glycine, a pH of 8.6 ± 0.2, and 20% methanol. The non-specific binding sites of the membrane were blocked with 0.2 g/10 mL of bovine serum albumin, 0.5 g/10 mL of non-fat dry milk, and 0.2% Tween-20 in PBS (phosphate-buffered saline).

For the “anti-HA” immunoblot, the primary antibody was mouse monoclonal anti-HA IgG1, #26183, clone 2–2.2.14, Thermo Scientific, RRID: AB_10978021. For the anti-ubiquitin (“anti-Ub”) immunoblot, the primary antibody was mouse monoclonal antibody type IgG1, raised against ubiquitin-conjugated lysozymes, #MA5-45328, clone FK2, Invitrogen/Thermo Scientific, RRID: AB_2931782. For the “anti-His” immunoblot, the primary antibody was mouse monoclonal anti-His_6_ IgG2b, #MA1-21315, clone HIS.H8, Invitrogen/Thermo Scientific, RRID: AB_557403. The primary antibodies were applied at a 10,000× dilution in PBS containing 10% blocking buffer for 1 h at room temperature. The secondary antibody (goat-anti-mouse IgG (H + L), horseradish peroxidase conjugate, R-05071-500; Advansta, Menlo Park, CA, USA, RRID: AB_10718209) was applied for 1 h under conditions similar to those of the first antibody. The membrane was washed once after the blocking phase and four to six times for 5–10 min in 20–30 mL of PBS containing 0.1% Tween-20 after the antibodies. The bands were visualized by the enhanced chemiluminescence detection method (WesternBright ECL HRP, Advansta) according to the manufacturer’s instructions.

The degree of dissociation of the TRESK protein to monomers varied between the experiments; the channel also migrated on the SDS-PAGE gel as dimers or higher-order aggregates. Attempts to improve the dissociation, e.g., boiling the sample, or the application of other detergents than NP-40 resulted in the complete loss of the signal. In some experiments, the blotting membrane was stripped and reprobed with another primary antibody. Stripping was performed for 45 min at 50 °C in a solution containing the following: Tris (62.5 mM, pH of 6.8, with HCl), SDS (2%), and mercaptoethanol (0.8%), and the membrane was washed six times for 10 min in PBS with 0.1% Tween-20.

### 4.6. Confocal Microscopy

Three days after the microinjection of HA_2_-N70Q-hTRESK with or without NDFIP1 cRNA, the oocytes were fixed for 30 min at room temperature in 4% paraformaldehyde dissolved in XenPBS, which contained 100 mM NaCl and 10 mM phosphate, with a pH of 7.4 (NaOH). The plasma membrane of the cells was permeabilized with Triton X-100 (0.3%) in XenPBS for 10 min. The non-specific binding sites of the fixed cell surface were blocked for 1 h at room temperature in 0.5 mL XenPBS containing 0.2 g/10 mL bovine serum albumin (BSA). The primary antibody was the same as in the anti-HA immunoblot reaction (see Section 4.5); however, it was diluted 1000× in 0.5 mL XenPBS containing 0.02 g/10 mL BSA. The secondary antibody was chicken anti-mouse IgG (H + L) cross-adsorbed secondary antibody, conjugated to Alexa Fluor^TM^ 488 (#A-21200, Thermo Scientific, RRID: AB_2535786) at a 1500× dilution in 0.5 mL of XenPBS containing 0.02 g/10 mL BSA. The cells were washed three times in 1 mL of XenPBS after the fixation, once after the permeabilization, once after the blocking phase, and three times for 5 min after the primary antibody. After the secondary antibody, the oocytes were washed six times for 5 min in 1 mL of XenPBS containing 0.2% Tween-20. The cells were examined by a Nikon A1plus (Nikon, Tokio, Japan), Ti2 Eclipse confocal microscope by using the NIS-Elements software (5.30.05).

### 4.7. Statistical Analysis

The data are expressed as the mean ± S.D. The statistical difference was considered to be significant at *p* < 0.05. Since the data appeared to deviate from a normal distribution and were heteroscedastic, a non-parametric analysis was used, a Kruskal–Wallis ANOVA followed by Dunn’s post hoc test for the comparison of mean ranks in multiple groups, or the Mann–Whitney U-test for two groups. The statistical calculations were performed in SPSS Statistics 29.0 (IBM Corporation, Armonk, NY, USA).

## Data Availability

The data are contained within the Results section of this article. Additional data are available from the corresponding author upon request.
